# Missing the Unhealthy? Examining Empirical Validity of Material Deprivation Indices (MDIs) Using a Partial Criterion Variable

**DOI:** 10.1007/s11205-016-1483-2

**Published:** 2016-11-23

**Authors:** Selcuk Beduk

**Affiliations:** Department of Social Policy and Intervention, University of Oxford, 43 Woodstock Road, Oxford, OX2 6HG UK

**Keywords:** Measurement validity, Unmet need for healthcare, Poverty measurement with multiple deprivation indicators, Disability and chronic health problems, Conversion factors, Multidimensional measurement of poverty

## Abstract

This study investigates the empirical validity of the material deprivation indices (MDIs) using a partial criterion variable, namely UHCNIR (unmet health care need due to inadequate resources). This alternative approach helps to assess absolute validity (Type I and II errors) and sources of error in the measurement of poverty for a specific aspect of poverty (in this case inability to receive adequate health care due to affordability problems). A simple mismatch analysis identifies a sizable group, around 1% of the adult EU population, missed by MDIs despite being in UHCNIR. A majority of this 1% experiences not only UHCNIR but also multiple other deprivations, commonly reports having some difficulties making ends meet, and prevalently has a disability or a chronic health problem. The analysis reveals that MDIs miss specifically those “unhealthy poor” since these measures do not include a relevant item, and thus cannot adjust for different needs and costs in health care and account for the distinct poverty experiences of these people. Therefore, the main methodological assumption of MDIs, identifying the people in poverty with only a limited set of key deprivation indicators is not supported by this empirical analysis.

## Introduction

There is now widespread agreement on the need to broaden the analysis of poverty beyond income-based assessments. Employing different methods, various poverty measures using multiple deprivation indicators have recently been proposed (e.g. Kakwani and Silber 2008; Betti and Lemmi 2013; Alkire and Santos 2013). Material deprivation indices (MDIs) are one example of such measures, now widely used to assess poverty in the EU. For example, 9-item MDI proposed by Guio (2009) is a formal measure to monitor the EU poverty target of lifting at least 20 million people out of poverty by 2020. Similar MDIs for the EU are proposed by Nolan and Whelan (2011), Guio et al. (2012) and Whelan and Maître (2012).

The primary aim of these indices is to identify individuals and groups in poverty. Yet recent critiques on the “multidimensional” approach point at possible validity problems—a break between concepts and measures, and thus an inconsistency between who is defined and measured as “poor”. Despite the influential concepts in the multidimensional approach, Nolan and Whelan (2011: 5) argue, “the linkage from concept through to application has often been weak and implementation rather *ad hoc*”. This separation of concepts from measures is partly due to data limitations but also reflects the level of theoretical and methodological advancement in the multidimensional approach. Ravallion (2011) claims that the design of multidimensional indices of poverty is usually data-driven and rarely rooted in a prevailing theory or grounded in robust methodological assumptions. These “rarely justified or critically scrutinized” methodological assumptions may however affect the accuracy of measurement (Chiappero-Martinetti and Von Jacobi 2012: 92).

The aim here is to assess whether such validity problems are relevant to the MDIs, and, if so, why. The paper examines such problems focusing on the concept of *empirical validity*, which evaluates the performance of the operationalization step—whether a concept is adequately translated into a measure.^1^

The existing evidence on the empirical validity of MDIs is limited. Studies usually focus only on construct validation and present “relative” evidence based on correlations. These studies claim validity by relating the newly proposed MDIs to the existing poverty measures (e.g. income poverty measures or previous MDIs) and comparing their ability to (1) identify high poverty-risk groups (e.g. low social class, unemployed, lone parents) (Nolan and Whelan 1996; Layte et al. 2001; Guio and Marlier 2013); (2) correlate most highly with the variables that are a priori expected to be associated with poverty (e.g. financial stress, psychological distress, low life satisfaction, bad health and various other welfare outcomes) (Nolan and Whelan 1996, 2011; Layte et al. 2001; Halleröd and Larsson 2008; Hick 2014, 2016); (3) explain within and between country differences in variables such as financial stress (Whelan et al. 2001; Whelan 2007; Whelan and Maître 2007, 2013); and (4) produce poverty rates consistent with social class and welfare regimes profiles (Nolan and Whelan 2011).

Focusing only on this method of construct validation, these studies are always and necessarily relative—evaluating the validity of measures against the validity of other measures (*relative validity*). However, what is more essential to the study of empirical validity is the evidence on Type I and II errors (*absolute validity*), and the possible sources of such error.^2^

Such evidence can be explored using *partial* criterion variables. A partial criterion variable is a standard that can qualify *some* individuals as poor (or non-poor).^3^ Despite focusing on a specific aspect of poverty, it is a sufficient criterion for poverty identification of certain groups (*partial criterion variable assumption*), hence can be used to identify some Type II errors. These Type II errors qualify as a significant validity problem if they (1) reflect systematic misclassification of certain groups, and (2) arise due to a problem in the design of MDIs. Therefore, using a partial criterion variable, the analyses focus on identifying some groups with certain characteristics whom are systematically missed by the MDIs.

Based on the poverty definitions employed in relevant studies, “unmet health care need due to inadequate resources” (*abbreviated as UHCNIR hereafter*) is suggested as a suitable partial criterion variable for poverty. If an individual cannot receive necessary health care because she/he cannot afford it, she/he might be considered as poor.^4^ Existing evidence suggests that UHCNIR more likely identifies those with lower resources and with a disability or a chronic health problem (Koolman 2007; Huber et al. 2008; Litwin and Sapir 2009; Bremer 2014). Moreover, MDIs might specifically miss these groups as they do not include relevant items, hence cannot adjust for varying needs and costs of health care.

Using UHCNIR as a partial criterion variable, the analysis therefore examines whether MDIs systematically miss those with lower resources and a disability or a chronic health problem; and, whether this is due to a structural problem in the design of MDIs.

To this end, firstly, Type II error is identified by a mismatch analysis between various MDIs and UHCNIR using simple cross tabulations. Secondly, the partial criterion variable assumption and the arguments on the sources of error are empirically investigated by examining the profiles of the missed group (those in UHCNIR but missed by MDIs) based on some health indicators and various other poverty measures (financial strain, social activities and comfort, subjective income inadequacy). Thirdly, the arguments on the significance and sources of error are formally tested in a regression model where MDIs are compared to two other poverty measures in their ability to estimate the criterion variable equally across health groups.

In the following sections, MDIs are described and the hypotheses on why MDIs might specifically be missing unhealthy poor is elaborated; data and methodology is described; UHCNIR is defined, and theoretically and empirically justified as a partial criterion variable for poverty. The article concludes with a discussion of the results and their implications on poverty measurement.

## What are MDIs?

 Material deprivation indices (MDIs) are single summary scales based on multiple deprivation indicators designed with the primary aim of improving the identification of poverty. The MDIs analyzed in this paper are described in Table 1. Despite employing a typical set of items from the EU-SILC survey, the MDIs differ in various aspects. This paper focuses on two approaches: the *consistent poverty approach* and the *consensual approach.* Originating in Townsend’s relative deprivation theory, the two approaches use similar but distinct definitions of poverty. Differences in definition then translate into differences in operationalization.

**Table 1:** Material deprivation indices of poverty

Guio (2009)	Guio et al. (2012)	Nolan and Whelan (2011)	Whelan and Maître (2012)
1. To pay their rent, mortgage or utility bills; 2. To keep home adequately warm; 3. To face unexpected expenses; 4. To eat meat or proteins regularly; 5. To go on holiday; 6. A television set; 7. A washing machine; 8. A car; 9. A telephone	1. To pay their rent, mortgage or utility bills; 2. To keep home adequately warm; 3. To face unexpected expenses; 4. To eat meat or proteins regularly; 5. To go on holiday; 6. A car; 7. PC & internet 8. Replace worn-out furniture 9. Some new clothes 10. Two pairs of shoes 11. Some money for oneself 12. Leisure activities 13. Drink/meal monthly	1. To pay their rent, mortgage or utility bills; 2. To keep their home adequately warm; 3. To face unexpected expenses; 4. To eat meat or proteins regularly; 5. To go on holiday; 6. A car; 7. PC	1. To keep their home adequately warm; 2. To eat meat or proteins regularly; 3. Replace worn-out furniture 4. to go on holiday; 5. Some new clothes 6. Two pairs of shoes 7. Some money for oneself 8. Leisure activities 9. Drink/meal monthly
*Thresholds*			
3+: Material deprivation (MD) 4+: Severe material deprivation (SMD)	5+: Material deprivation (MD) 7+: Severe material deprivation (SMD)	2+: Material deprivation (MD) 3+: Severe material deprivation (SMD)	2+: Material deprivation (MD) 3+: Severe material deprivation(SMD)
*Operationalization*			
Consensual criterion Expert criterion	Consensual criterion Expert criterion	Expert criterion	Expert criterion
*vis-à-vis income poverty measures*			
Union method Intersection method	Union method Intersection method	Intersection method	Intersection method
*Approach*			
Consensual approach Consistent poverty approach	Consensual approach Consistent poverty approach	Consistent poverty approach	Consistent poverty approach

The older MDIs, Guio (2009) and Nolan and Whelan (2011), employ items from the main EU-SILC survey, while the updated versions, Guio et al. (2012) and Whelan and Maître (2012), utilize the special module survey on material deprivation. The data on items such as clothes, shoes, leisure activities and spare money are collected in the special module and therefore does not exist for the older indices

Conceptualized by Ringen (1985, 1988) based on an interpretation of Townsend’s definition and applied *inter alia* by Nolan and Whelan (1996, 2011), the *consistent poverty approach* defines poverty as “exclusion from society due to inadequate resources”. This concept of exclusion is closely related to the Townsend’s notion of “inability to participate in ordinary living patterns”. For operationalization, Nolan and Whelan (1996) focus on specifying what *manifests* these ordinary living patterns and identify related deprivation items based on some criteria derived from the definition and some practical policy considerations (*expert criterion*).

Originally developed by Mack and Lansley (1985, 2015) based on a fundamental conceptual critique of Townsend (1979) and applied and advanced *inter alia* by Gordon (2006) and Guio (2009), Guio et al. (2012), the c*onsensual approach* defines poverty as “exclusion from a minimally accepted way of life due to inadequate resources”. For operationalization, Mack and Lansley (1985) focus on identifying what *constitutes* this minimum level of living, and apply an attitudinal survey to specify socially perceived necessities for having a minimally accepted way of living (*consensual criterion*).

Another distinction between these two approaches relates to their use vis-à-vis income poverty measures. The MDIs of the consensual approach are usually employed following a *union method*—an individual or a household is counted as “poor” if identified by either a MDI or an income poverty measure; the total poverty is the union of the two. The MDIs of consistent poverty approach are usually employed following an *intersection method*—an individual or a household is identified as “poor” if identified by both an MDI and an income poverty measure.

Applying this framework, the MDIs proposed by Nolan and Whelan (2011) and Whelan and Maître (2012) are readily distinguished as measures of consistent poverty approach. Nevertheless, the indices proposed by Guio (2009) and Guio et al. (2012) are hybrids using Townsend and Mack and Lansley, with indicators mainly based on consensual criterion and expert criterion. Despite reflecting an inconsistency, the hybrid nature of these pragmatic measures means that both the union and the intersection methods may be used in their implementation as Guio (2009) has also done.

### Possible Sources of Error for MDIs

A measure lacks empirical validity when significant measurement error occurs. Measurement error has two components: systematic and random. The focus here is on the (non-survey) systematic error that occurs when the concepts are not fully reflected by the measures due to problems of design. Given limited data, certain methodological assumptions made in the design of MDIs might be empirically unfounded and be the actual sources of systematic error. This is especially possible when the specification of MDIs is guided not by theory but by data.

Studies of MDIs usually select relevant deprivation items based on theory but construct indices based on data-driven methods (exploratory factorial techniques). Hence, the specification of resulted scales is ultimately data-driven. Yet the development of these data on deprivation items, in ECHP or EU-SILC, has been fairly arbitrary, “different countries learning from each other while having their own preoccupations” (Nolan and Whelan 2011:15). The available deprivation items therefore do not represent a coherent conceptual framework appertaining to poverty. Indeed, the limited range of items in the EU-SILC means that any index constructed from these items will be inadequate to capture the concept of poverty as exclusion from ordinary living patterns (Berthoud and Bryan 2011; Maître et al. 2013: 5; Hick 2014). As a result, the MDIs necessarily include only *some* key indicators but do not cover all different aspects of poverty (Guio et al. 2012).

The main methodological assumption of MDIs is that a single scale based only on some key deprivation items is adequate to identify people in poverty. But recent multidimensional applications have shown that accounting for the joint distribution of all dimensions is not only useful for understanding the varying patterns of distinct dimensions but also a determinant of who is identified as “poor” (Whelan et al. 2014; Alkire et al. 2015; Hick 2016). This raises concerns about the validity of the summary MDIs. If items relating to certain relevant need and deprivation structures are not included, the MDIs might fail to identify related cases of poverty.

One such aspect of poverty that is not accounted for in MDIs is health care. Extra costs of disability and the need for equivalization across households with different health conditions have long been realized in the literature on income poverty (Mayer and Jencks 1989; Sen 1992; Zaidi and Burchardt 2005; Morciano et al. 2015).^5^ The same equivalization problem applies to MDIs as they do not include relevant items, and thus cannot adjust for the differences in health care needs and costs.

MDIs typically include items from different types of poverty such as food, fuel, housing, durables, financial strain and social activities. These different dimensions are usually combined into a single scale of poverty, and a threshold on the total number of deprived items is specified to distinguish poor from non-poor (counting approach). Yet one threshold on a single scale including different types of deprivations might not be adequate to capture a multidimensional phenomenon such as poverty. Different need patterns might translate into different thresholds in a single scale. So, healthy and unhealthy groups might have different thresholds in a single scale which does not account for the differences in needs and costs for health care. As a result, MDIs might miss those individuals with worse health conditions whose higher health care expenses might pull down their already low living standards to the state of poverty.

## Data and Methodology

### Data

This paper uses the 2009 wave of EU-SILC survey including a special module on material deprivation. The sample provides information on EU-27 countries as well as Norway and Iceland. Given that the focus is on the EU, Norway and Iceland are excluded. The unit of analysis is individual. Hence most information on deprivation is collected at the household level is allocated to each household member. However, information on some deprivation items (e.g. clothes, shoes, and social activities), disability, chronic health and subjective general health are collected only for the household reference person (HRP) in the ‘register” countries using administrative data such as Denmark, Finland, Netherlands, Sweden and Slovenia.^6^

One way to deal with this problem is to allocate the HRP information to other household members, as adopted by Guio et al. (2012). This might be a good strategy for the deprivation items given the assumption of intra-household transfers. However, the same assumption might be harder to justify for UHCNIR as the unmet need question includes individuals’ own assessment of their health. Also, low levels of UHCNIR in these register countries do not provide much variance for the analysis. Therefore, these five register countries (7% of the total sample) are excluded from the analysis.^7^ To have a balanced sample, missing cases lower than 1% for the variables of interest are excluded from the sample.^8^ The final sample size contains 349,438 individuals and represents a target population of adults (16+) in 21 EU countries.

The construction of the criterion variable as well as other measures used in the analysis is described in Table 2 below. Two variables are used to construct UHCNIR. The first one asks each adult individuals whether they have unmet needs for medical examination or treatment. If one answers yes, a follow-up question asks the specific reasons for this unmet need—is it because of waiting lists, too far to travel/no means of transportation, could not take time because of work, care for children (among other reasons), or is it actually because they could not afford to? The UHCNIR rate is calculated as the ratio of those “having unmet health care need because they could not afford to” to the total sample.

**Table 2:** Description of variables used in the analysis

Variable	Survey question/Description	Scale	Recode
UHCNIR—unmet health care need due to inadequate resources	a. “Was there any time during the last twelve months when, in your opinion, you personally needed a medical examination or treatment for a health problem but you did not receive it?” b. (for those positively answer question a) what are the reasons for this unmet health care need? *derived using questions a and b*—answering yes to the question a and stating “could not afford to” for the question b	a. 1: yes at least one case 0: no b. multiple categories from 1 to 8^a^ Binary—0/1	
Subjective health	“How is your general health?”	Interval 1 to 5 1: Very good 5: Very bad	Binary—1 if SH > 3 0 otherwise
Disability	“For at least the past 6 months, to what extent have you been limited because of a health problem in activities people usually do? “	Binary—0/1	
Chronic health problem	“Do you have any longstanding (6 months or more) illness or health problem?”	Binary—0/1	
Bad health	*derived*—reporting bad subjective health or disability or chronic health problem	Binary—0/1	
SMD index (EU’s formal pov. measure)	*derived*—9-item index of Guio (2009)	Binary—1 if SMD > 3; 0 otherwise	Interval—0 to 9
Relative income poverty (EU’s formal pov. measure)	*derived*—Having equivalized household income below 60% of median household equivalized income	Binary—0/1	
Work intensity/joblessness (EU’s formal pov. measure)	*derived*—Eurostat definition	Binary—0/1	
EU 2020 poverty headline indicator (at-risk-of-poverty and social exclusion)	*derived*—Being identified as “poor” by SMD index *or* Income poverty *or* joblessness measures	Binary—0/1	
(Subj.) income inadequacy	“Thinking of your household’s total income, is your household able to make ends meet, namely, to pay for its usual necessary expenses?”	Interval 1 to 6 1: With great difficulty 3: With some difficulty 6: Very easily	*Binary form I:* 1 if SII < 3 0 otherwise *Binary form II:* 1 if SII < 4 0 otherwise

^a^1: could not afford to (too expensive), 2: waiting list, 3: could not take time because of work, care for children etc., 4: too far to travel—no means of transportation, 5: fear of doctor—hospitals—examination—treatment, 6: wanted to wait and see if problem got better on its own, 7: didn’t know any good doctor or specialist, 8: other

### An Alternative Methodological Approach to Test Empirical Validity of MDIs

In the existing literature, validity evaluations focused on the construct validation method. As reviewed above, these studies typically examine whether their new MDIs vis-à-vis the existing poverty measures are more closely related to the variables that are a priori expected to be related (e.g. various welfare problems or risk groups). For these empirical tests, there is no rule of thumb for interpreting the size of a correlation or an odds ratio. Construct validation therefore necessarily provides evidence only on relative validity. However, to establish absolute validity of a measure requires knowledge of both the number of people erroneously identified as being in poverty (Type I) and the number of people experiencing poverty who were missed by the measure (Type II). Also, existing validity evaluations are usually undertaken primarily to justify the merits and properties of newly proposed measures. Yet, validity evaluations are, in general, performance assessments, useful for identifying areas of improvement. However, there is not much evidence and discussion on the *sources* of error in the literature.

To facilitate such discussion on the absolute validity and sources of error, an alternative approach that can detect Type I and II errors is necessary. The question to be explored is the following: whatever the type or form of poverty, are the MDIs able to identify if it is a case of poverty? Different types or forms of poverty might include a set of dimensional deprivations or a set of item deprivations. Yet it might not be readily clear from the existing definitions which sets of deprivations imply a case of poverty. For example, how many and which combinations of deprivations can be held to define the poverty experience?

Given the difficulty of specifying *each* set of deprivations that entail a case of poverty, the goal here is to concentrate on *a specific* type of deprivation that is defined to be a component of the poverty experience, but not included in the MDIs. An indicator of such deprivation can be described as a partial criterion variable. Such criterion variable can benchmark certain cases of poverty. Then a simple mismatch analysis between the MDIs and the criterion variable can be used to identify some Type II error related to a certain aspect of poverty.

One objection to that might be the insufficiency of one dichotomous variable to identify poverty. Individuals might not change their way of living due to a single deprivation by utilizing their savings or social support mechanisms (Callan et al. 1993; Mack and Lansley 1985; Edin and Lein 1997). This view rejects an understanding of poverty as a multi-component experience and thus looks for the evidence for multiple simultaneous deprivations. To investigate this and justify UHCNIR as a partial criterion variable, firstly, the theoretical and empirical relationships of UHCNIR and poverty are investigated; and secondly, the profiles of the people missed by the MDIs (despite being in UHCNIR) are examined using multiple other poverty measures, including a financial strain index proposed as a validity measure by Maître et al. (2012).

Also, with the proposed criterion variable, only Type II error can be evaluated. The proposed criterion variable is partial, focusing on a specific aspect of poverty rather than the whole domain. So, while being deprived of the criterion variable indicates poverty, not being deprived of the criterion variable does not necessarily indicate a non-poverty state—individuals might be experiencing multiple deprivations other than the criterion variable. Therefore, the proposed partial criterion variable is reliable only for examining Type II errors.

Yet, the identified Type II error for a specific aspect of poverty does not readily amount to a significant measurement error. To qualify as a substantial empirical validity problem, the total Type II error should either result in significant over or under-estimation, or cause systematic misclassification of certain groups. Given its partial nature, the analysis here cannot examine the total error in poverty incidence. If the error is random, some Type I error might cancel out the identified Type II errors, making the overall incidence unaffected. Also, errors related to other aspects of poverty cannot be evaluated based on a partial criterion variable. Therefore, such analysis based on a partial criterion variable does not provide an evaluation of the size of total error. Still, a careful selection of the partial criterion variable can help to identify some systematic error of *misclassification of certain groups*.

UHCNIR is an indicator that specifically reflects problems related to health care access, and captures those who have lower resources and higher health care needs (e.g. individuals with a disability or a chronic health problem). So, a significant disagreement between MDIs and UHCNIR can show that MDIs specifically miss some “unhealthy poor”. This is inspected by examining the profiles of the missed group based on some economic and health indicators.

This misclassification is a significant validity problem only if it is systematic, due to a problem in the design of the measures. Comparing the performances of MDIs to other poverty measures can provide evidence on the sources of error. MDIs might fail to identify those people in poverty with a disability or a chronic health problem due to their inability to account for need differentials in health care. If so, a relative income poverty measure also cannot adjust for the differences in health care needs and then would also suffer from the same problem; however, an income inadequacy measure can adjust for need differentials (as individuals evaluate their overall resources against needs), and can equally identify healthy and unhealthy groups.

These hypotheses are formally tested in a regression setting. If a measure systematically misses the “unhealthy poor”, it cannot predict the partial criterion variable equally (with the same precision) across different health groups, but predict worse for the unhealthy groups. Then, MDIs and relative income poverty measures are expected to estimate worse for the unhealthy groups; on the other hand, a subjective income inadequacy measure can estimate the criterion variable equally across health groups. The idea is to compare the ability of poverty measures on predicting the partial criterion variable across different health groups; in other words, testing the interaction effect of health status on the relationship between poverty measures and the criterion variable.

For an initial investigation of this, the odds of agreement between UHCNIR and poverty measures are compared across different health groups using Mantel–Haenszel method. Given all measures are binary, in a 2 × 2 cross tabulation, the cell frequencies “*a*” and “*d*” show the agreement, and “*b*” and “*c*” show the disagreement between the criterion variable and the poverty measure (Table 3).

**Table 3:** Cross tabulations of poverty measure and criterion variable

	Partial criterion variable (UHCNIR)	
	0	1
Poverty measure (e.g. MDI)		
0	a	b
1	c	d

*Pr(agreement)* (a/n) * (d/n), *Pr (disagreement)* (b/n) * (c/n), *Odds ratio* (a*d)/(b*c)

a, b, c, d are cell frequencies. n is the total sample size

Then, the odds ratios are calculated as the ratio of probability of agreement (a*d) to the probability of disagreement (b*c).^9^ The null hypothesis is the homogeneity of odds ratios across health groups examined based on a Chi square test. We expect to reject the null for MDIs and relative income poverty measure but not for the income inadequacy measure.

To test the interaction effect of health status, variants of the following model are estimated for an MDI, a relative income poverty measure and a subjective income inadequacy measure.

$$logit\left( {{\text{UHCNIR}}_{\text{i}} } \right) =\upalpha +\beta_{1} {\text{P}}_{\text{i}} +\beta_{2} {\text{H}}_{\text{i}} +\beta_{3} {\text{P}}_{\text{i}} *{\text{H}}_{\text{i}} +\beta_{4} {\text{C}} + {\text{e}}_{\text{i}}$$

The dependent variable, UHCNIR is the criterion variable; P is a binary poverty measure (e.g. SMD index); H is a binary health indicator (1 = bad health); P*H is their interaction term and C represents the controls such as age.^10^

To examine the significance of the interaction terms, firstly, the models are run with and without the interaction term for each poverty measure, and the deviance between the models are examined using likelihood-ratio test. The null hypothesis is equal likelihood between the models with and without interaction term. Then, we expect to reject the null hypothesis only for MDIs and relative income poverty measure but not for the income inadequacy measure.

Secondly, the significance and the direction of the estimated coefficients of the interaction terms are examined. In the proposed model, coefficient β represents the predictive power of a poverty measure (P). If the interaction term is significant, the predictive power of the poverty measure for unhealthy individuals is β + δ, while it is β for healthy individuals. If the interaction term is *significant* and *negative* (in odds ratio terms, a coefficient term lower than one), the poverty measure estimates worse for the unhealthy group. Then, a significant and negative interaction term in the models of MDIs and the relative income poverty measure, and a non-significant interaction term in the model of the income inadequacy measure are expected.

## UHCNIR: A Solid Partial Criterion Variable for Poverty?

The indicator of unmet health care need has recently been proposed as a new indicator for assessing access to health care (Koolman 2007; Allin and Masseria 2009). Unmet need arises when an individual does not receive an available and effective treatment. Allin et al. (2010) define unmet need for health care as a multidimensional concept, distinguishing five different kinds or categories of unmet need: (1) *unperceived unmet need*, where an individual does not recognize her need for health care; (2) *subjective*, *chosen unmet need*, where an individual perceives himself as in health care need but does not demand the services available; (3) *subjective*, *not*-*chosen unmet need*, where an individual perceives herself as in some kind of health care need but does not receive services due to some access barriers beyond her control, (4) *subjective*, *clinician validated unmet need*, where an individual perceives himself a need for health care and accesses health care which validates his perception, but does not receive a treatment that a clinician would judge as appropriate, (5) *subjective unmet expectations*, where an individual perceive herself as in need for some kind of health care, receives care but in her view does not constitute a suitable treatment.

Given its relation to poverty, this paper focuses only on the third type of unmet need (using the follow-up question asking the respondents their reasons of unmet need). If an individual does not receive a medical examination (e.g. a doctor’s visit) or treatment (e.g. drugs, surgery) specifically because she cannot afford them, she experiences *subjective*, *not*-*chosen unmet need that occurs beyond her control* (Allin et al. 2010). There are various terms used in the literature such as cost-related medical non-adherence, foregone care, underutilization or underuse and cost barriers (Litwin and Sapir 2009). The term that is used in this study related to the survey question is “unmet health care need due to inadequate resources” (UHCNIR).

Such deprivation can be justified as a component of poverty given definitions used in relevant studies. Definitions of both consistent and consensual approaches can be categorized into two common main parts (Nolan and Whelan 2007): “exclusion from society” and “inadequate resources”. The latter is the *enforced criterion*—a deprivation of exclusion caused by a lack of economic resources and not by other reasons (e.g. preferences). For the former, approaches differ in their conception of “exclusion from society” (ordinary vs. minimum): Townsend (1979) focuses on a norm or an ordinary living pattern defined based on “objective” individual needs, while Mack and Lansley (1985) focuses on a minimum level based on “subjective” (and consensual) societal needs. *Therefore*, *a suitable partial criterion variable should be a deprivation which is caused by a lack of resources (enforced criterion)*, *a part of accustomed living standard in the EU society (based on expert criterion) and be perceived by the majority of EU citizens as a socially perceived necessity (based on consensual criterion).*

In this context, having problems of access to health care (due to a lack of resources) can be considered as a component of the poverty experience for both approaches. In the EU, access to health care has long been one of the building blocks of the welfare states. Now almost at a universal level, citizens are covered by health care insurance which enables their access to health care services and protects them against the financial impacts of unexpected health problems (OECD 2014). Also based on a special Eurobarometer survey, 77% of the people in the EU consider having “medical care when needed” as *absolutely* necessary for a minimally accepted living standard, the highest support among all other items (Dickes et al. 2010).^11^

However, despite the achievement of universal coverage for a comprehensive set of health care services in the EU, there are still reported problems of access (Doorslaer et al. 2004; Allin and Masseria 2012).^12^ For example, in 2009, 4.2% of the EU 27 population reports having unmet health care needs, and approximately half of these are due to affordability problems.^13^ The literature reports the key determinants for these access problems as the out-of-pocket payments and direct costs of care to patients (Lostao et al. 2007; Or et al. 2008; Litwin and Sapir 2009; Devaux and De Looper 2012; Bremer 2014).

Despite being perceived as a basic need and an ordinary part of living in an EU society, a small but significant group of people cannot access certain health care services because they cannot afford the direct costs of care. Therefore, having UHCNIR can be considered as living below a minimally accepted living standard or not being able to participate in ordinary living patterns in the EU. Moreover, the UHCNIR indicator complies with the enforced deprivation criterion as it evaluates whether the deprivation is caused by a lack of economic resources. So theoretically, the UHCNIR indicator can be a good partial criterion variable for poverty for both consensual and consistent poverty approaches.

This can also be justified based on some empirical evidence showing a close relationship between UHCNIR and poverty (measured with the formal EU 20202 headline poverty measure). *Firstly*, there is a very high cross-country correlation for the EU countries between UHCNIR and poverty (0.86). *Secondly*, the risk profiles of UHCNIR and poverty are almost the same (see Table 4 and also “Appendix”). A specific sub-group is defined to be at risk if the ratio of the group among the people in UHCNIR (or poverty) is higher than the ratio of the group in the total population. For example, the ratio of unemployed among people in UHCNIR (14.45%) is significantly higher than the ratio of unemployed in total adult population (6.09%).

**Table 4:** Risk groups for UHCNIR and poverty

	UHCNIR	EU2020 poverty indicator
Gender	Female	Female
Age group	45–64, 65+	15–24, 45–64
Household type	Single person, Single parent, Extended family	Single person, Single parent, Extended family
Marital status	Widowed, Divorced	Never married, Widowed, Divorced
Place of residence	Thinly populated	Thinly populated
Economic status	Unemployed, Retired, Disabled, Unpaid domestic worker, Other inactive	Unemployed, Student, Disabled, Domestic unpaid worker, Other inactive
Occupation	Service workers, Skilled agr. and fishery worker, Craft trade workers, Elementary occupations	Service workers, Skilled agr. and fishery worker, Craft trade workers, Elementary occupations
Health	Fair, bad, very bad reported health, Disability, Chronic health problems	Fair, bad, very bad reported health, Disability, Chronic health problems

The risk groups for UHCNIR (or poverty) are identified as the groups whose ratio within the people in UHCNIR (poverty) is higher than their ratio within the population. Data is presented at the Appendix

*Thirdly*, risk profiles reflect only bivariate associations, but even after conditioning the probability on other factors in a logit model, the close relationship between UHCNIR and poverty remains strong. After controlling for income, gender, age, marital status, and health problems (subjective health, disability and chronic health problems), the people in UHCNIR are three times more likely to be in poverty than others (see “Appendix” for results). These results provide a certain level of confidence in using UHCNIR as a criterion variable for poverty.

## Results: Examining Empirical Validity of the MDIs

As described in Table 1, four main MDIs with their different variants are examined in the mismatch analysis. Two error statistics for Type II error, TIIE_1_ and TIIE_2_, are calculated based on the cross tabulations of MDIs and UHCNIR. To illustrate the data and calculation of the statistics, raw results for Guio (2009)’s SMD index (4+) are presented at Table 5 below.

**Table 5:** Mismatch analysis with a criterion variable of UHCNIR

		UHCNIR		
		0	1	
SMD index	0	90.6%	**1.05%**	91.7%
	1	7.36%	0.99%	8.34%
		97.97%	2.03%	100%

Bold shows the proportion of people in UHCNIR but not identified by the SMD index—Type II error

The results show a majority, around 90%, is neither in severe material deprivation nor in UHCNIR. Around 1% is identified by both measures; around 7% suffers only from SMD and around 1% only from UHCNIR. This latter group of 1% indicates the TIIE_1_ statistic: 1% of the population is in UHCNIR but not identified by the SMD index. The TIIE_2_ statistic equals to 1.05/2.03 = 0.52: the SMD index does not identify around 50% of the people in UHCNIR.

These error statistics are calculated for all measures as shown above in Table 6. For all MDIs, The TIIE_1_ statistics are usually above 1% (except the MD variants in consensual approach): around 1% of the EU adult population is missed by the MDIs despite being in UHCNIR. In other words, this 1%, corresponding to 3.5 million adults, might be wrongly identified by the MDIs as “non-poor”.^14^ Also, TIIE_2_ statistics for MDIs are around 0.50-0.75 (except the MD variants in consensual approach): more than half of the people in UHCNIR are not identified as “poor” by MDIs.^15^

**Table 6:** Type II error statistics for MDIs and other poverty measures

CONSENSUAL APPROACH Single MDI measures where indicators identified with a consensual method			
MDIs	TIIE_1_	TIIE_2_	Headcount rate%
Guio 2009—9 item			
3+ MD	0.006	0.31	17.4
4+ SMD	0.011	0.52	8.3
Guio et al. 2012—13 item			
5+ MD	0.006	0.29	20.2
7+ SMD	0.010	0.51	10.4

CONSISTENT POVERTY APPROACH MDIs combined with an income poverty measure (60% of national median equivalized household disposable income)			
Guio 2009—9 item			
3+ MD	0.014	0.67	6.6
4+ SMD	0.015	0.75	3.8
Guio et al. 2012—13 item			
5+ MD	0.014	0.68	7.2
7+ SMD	0.015	0.76	4.3
Nolan and Whelan 2011—7 item			
2+ MD	0.013	0.63	10.2
3+ SMD	0.014	0.67	6.8
Whelan and Maître 2012—9 item			
2+ MD	0.013	0.63	9.7
3+ SMD	0.013	0.66	7.3

*TIIE*_*1*_ Ratio of Type II error to total population, *TIIE*_*2*_ Ratio of Type II error to the # of people in UHCNIR

The error statistics are substantively high given the assumption that the people who experience UHCNIR also experience poverty. But, firstly, is this assumption tenable? In other words, does this 1% who is in UHCNIR but missed by MDIs really experience poverty? Secondly, for this error to be a significant validity problem, this 1% must reflect certain common characteristics and systematically be missed due to a structural problem in the design of MDIs. Hence, what is the source of error?

### Relaxing the Assumption of Partial Criterion Variable

To test the partial criterion variable assumption, hence further investigating the poverty status of this 1%, other poverty indicators can be observed. Table 7 below shows the results for the measures of relative income poverty, work intensity, subjective income inadequacy (form II), below 120% of median household income. A “financial strain” index^16^ proposed as a validity index by Maître et al. (2012), and a “basic comfort and activities” index^17^ are also examined. This analysis held only for the SMD index but the results are similar for other MDIs.

**Table 7:**
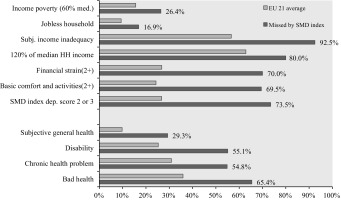
Does this 1% experience poverty?

The table gives rates of some poverty-related indicators for the 1% who are missed by the SMD index of Guio (2009) despite being in UHCNIR. The EU average numbers are the rates for the adult EU population. The financial strain rate indicates the ratio of people who are deprived of two or more items from the 5-item list of financial strain index (see Footnote 14). The basic comfort and activities rate indicates the ratio of people who are deprived of two or more items from the 5-item list of basic comfort, leisure and social activities index (see footnote 15)

As shown in Table 7, among these 1% missed by MDIs despite being in UHCNIR, around 26% is in income poverty and 17% lives in jobless households. Although these rates are significantly higher than the EU average (15.6% for income poverty and 9.4% for jobless households), majority of this 1% is still not in income poverty and does not have low work intensity. On the other hand, almost all of this 1% reports having inadequate income (93%); 70% reports two or more deprived items from the 5-item financial strain index (compared to an EU average of 26%); 70% reports two or more deprived items from the 5-item index on “basic comfort and social activities” (compared to an EU average of 24%); and, only around 10% does not report any deprivation out of these three deprivation indices.

Therefore, despite not being identified by the SMD index, *the majority* of this 1% not only experience UHCNIR but also suffer from multiple other deprivations and reports some difficulties making ends meet. Also, when the risk profiles of this 1% are compared to the poverty risk profiles explored before, they are exactly the same as in Table 4. Therefore in all relevant indicators, this group shows very similar characteristics to the people in poverty.

### Sources of Error

But despite not being jobless or in income poverty, why do these people significantly report having difficulties making ends meet? One reason for this might be extra costs related to having *higher health care needs*. The UHCNIR indicator reflects a condition of having inadequate resources relative to the needs for health care. The people identified by the UHCNIR typically have lower resources and higher health care needs (Koolman 2007; Allin and Masseria 2012). This is also valid for this 1%—around 80% of them fall below 120% of the median household income; also, around 29% reports bad subjective health, 55% has a disability or a chronic health problem, and around 65% suffers from either of these three. These are significantly high numbers compared to the EU average.

An individual with a disability or chronic health problem might need periodical medical checks or need to regularly take pharmaceuticals. These all means higher costs, for example, given the amount of co-payments or transportation expenses. Indeed, as referred before, the evidence suggests that the main factor explaining UHCNIR is the amount of out-of-pocket payments. Therefore, economic resources of this 1% might be adequate to put them above the relative income poverty thresholds despite still not being enough to meet their health care needs. Indeed, majority of this 1% locates just above the relative income poverty threshold—60% have incomes lower than the 4th decile and 80% is below the 6th decile or below 120% of median income. *Therefore*, *the issue of this 1% is not low income but inadequate income.*

MDIs also cannot fully account for the poverty experiences of the people with a disability or a chronic health problem because the scales are constructed without any consideration on the varying health care needs. MDIs do not include a relevant deprivation item that can adjust for these need and cost differentials. Despite suffering from lower resources and multiple deprivation, these people are not identified by MDIs since these people’s distinct need patterns translate into a different threshold in a scale where health care needs are not included. Indeed 74% of the people not identified by the SMD index are still deprived of two or three items from the SMD index, so locates just below the threshold of four, but still not identified as in poverty by MDIs.

### Testing the Arguments

The analysis showed that MDIs specifically miss a sizable group of unhealthy poor mainly due to their inability to adjust for need differentials in health care. If the MDIs miss specifically the individuals with worse health conditions, then the MDIs cannot estimate the criterion variable equally (with the same precision) for different health groups, and estimate worse for the “unhealthy”. Also, if the problem of MDIs is “need differentials”, a relative income poverty measure would suffer from the same problem of discriminating against unhealthy but income inadequacy measure would estimate the criterion variable equally across the health groups.^18^

To test these hypotheses, firstly the odds of agreement between poverty measures and UHCNIR are compared across the health groups using Mantel–Haenszel method (Table 8). (See “Appendix” for the cross-tabulations of poverty measures and UHCNIR across health groups).

**Table 8:** Odds ratios for different health groups

	Odds ratio	95% confidence interval	Test of homogeneity
SMD index			
Healthy	9.97	9.21–10.79	Chi^2^(1) = 7.19
Unhealthy	8.48	8.28–9.25	Pr > Chi^2^ = 0.0073
Relative income poverty			
Healthy	4.06	3.75–4.40	Chi^2^(1) = 27.97
Unhealthy	3.13	2.96–3.31	Pr > Chi^2^ = 0.000
Income inadequacy			
Healthy	18.5	14.78–23.45	Chi^2^(1) = 0.11
Unhealthy	17.6	14.95–20.86	Pr > Chi^2^ = 0.74

For all measures, the odds of agreement between the poverty measures and UHCNIR are higher for healthy compared to the unhealthy. For example, SMD index and UHCNIR are about 10 times more likely to agree than disagree for healthy groups, while this number is 8.5 for the unhealthy groups. However, the differences between the odds ratios of healthy and unhealthy groups are significant only for the SMD index (*p* = 0.007) and the relative income poverty measure (*p* = 0.00), but not for the income inadequacy measure (*p* = 0.74). These results show that associations of the SMD index and the relative income poverty to the criterion variable depend on the health status. The SMD index and the relative income poverty measure estimate the criterion variable better for healthy, while the income inadequacy measure predicts equally across health groups.

Secondly, to test the significance of interaction terms, six logistic regressions are run: models with and without interaction terms for each of the three poverty measures (model is presented in the methods section). The results are shown in Table 9 below. The log likelihoods, and deviance statistics and tests are also reported in the table.

**Table 9:** Testing the interaction effect of health status

	Model 1 UHCNIR OR/se	Model 2 UHCNIR OR/se	Model 3 UHCNIR OR/se	Model 4 UHCNIR OR/se	Model 5 UHCNIR OR/se	Model 6 UHCNIR OR/se
Guio’09 (*SMD*)	9.26*** (.21)	10.45*** (.401)				
SMD*bad health		.836*** (.043)				
Income poverty (IP)			3.50*** (0.08)	4.26*** (.165)		
IP * bad health				.751*** (.038)		
Income inadequacy (II)					18.15***	19.08*** (2.18)
II * bad health						.925 (.13)
Bad health	3.44*** (.09)	3.74*** (.130)	3.75*** (.10)	4.18*** (.130)	3.63*** (.10)	3.91*** (.55)
N	349,438	349,438	349,438	349,438	349,438	349,438
Log likelihood	−33,063.9	−33,057.3	−35,872.2	−35,855.5	−34,709.6	−34,709.4
Lrtest Chi^2^		13.34		33.97		0.30
Lrtest *p*		0.0003		0		0.58

The dependent variable for all models is UHCNIR. The results for the coefficients are presented in odds ratio terms. The results are the same if the models are ran separately for each health indicator. The model is presented for Guio (2009) but other MDIs give similar results and available upon request. The models include age as a control

The deviance between the models with and without interaction terms is significant for the models of SMD index (Model 1 and 2) (*p* = 0.0003) and relative income poverty (Model 3 and 4) (*p* = 0.00), while it is not significant for the income inadequacy (Model 5 and 6) (*p* = 0.58). This means adding the interaction term to the model makes a significant difference only for the SMD index and the relative income poverty measure.

In addition, the interaction terms are significant in model 2 and 4 but not significant in model 6. This shows that the predictive power of SMD index and relative income poverty depends on the health status of individuals while the income inadequacy measure predicts equally across health groups. Moreover, the coefficients of the interaction terms in model 2 and 4 are smaller than one, which means the SMD index and the relative income poverty measure predicts the criterion variable worse for the unhealthy groups. The predictive power of the SMD index is 17% lower for the unhealthy than for the healthy ones. The predictive power of the relative income poverty measure is 25% lower for the unhealthy than for the healthy ones. On the other hand, the interaction term in Model 6 is not significant and has a very low effect size, showing only a difference of 6.5% between the predictive power of income inadequacy for healthy and unhealthy groups. This shows the ability of income inadequacy measure to estimate equally for different health groups, and not missing specifically the unhealthy groups.

These evidences reinforce the finding of the mismatch analysis that the MDIs tend to miss the unhealthy poor. Moreover, this error is probably non-random and due to not accounting for the health care need differentials since (1) the relative income poverty measure suffers from the same validity problem even more than the SMD index, while (2) the income inadequacy measure estimate equally for different health groups.

## Conclusion

The study investigated the empirical validity of MDIs (from the health care aspect of poverty) and the sources of possible error. Given that existing correlational evidence based on construct validation does not provide an adequate basis for examining absolute validity, an alternative approach based on a partial criterion variable is employed. In this approach, UHCNIR (unmet health care need due to inadequate resources) is used as a criterion for identifying some people in poverty with a disability or a chronic health problem. Theoretically, this type of deprivation is a component of poverty given the definitions used in the relevant studies. Empirically, the indicator identifies mostly people with lower resources, and a disability or a chronic health problem, and highly correlates with and produces similar risk profiles to the formal poverty measures. A mismatch analysis between MDIs and UHCNIR then identifies those individuals who can be defined as poor but missed by MDIs—Type II error.

The results of the mismatch analysis show that the MDIs miss a sizable group of adults—3.5 million—who reports having unmet health care needs due to inadequate resources (UHCNIR). Despite not being identified by MDIs, three out of four of this 1% experiences multiple other deprivations in basic comfort and social activities, and in financial strain; most of them have limited resources to meet their needs (80% have income lower than 120% of median); and, almost all (around 90%) report having some difficulties making ends meet.

This Type II error is a significant empirical validity problem because it results in systematic misclassification of the unhealthy poor and is attributable to a limitation in the design of MDIs. Around three out of five missed by the MDIs has either a disability or a chronic health problem. MDIs specifically miss these people with some serious health problems because varying health care needs and costs are not taken into account in the design of these measures. For the same reason, the relative income poverty measure also misses the same group, but the subjective measure of income inadequacy do not suffer from the same problem as it by design adjusts for need differentials.

Lowering thresholds appears to be one solution to the validity problems identified. For all MDIs, lower thresholds (MDs) performed significantly better than higher thresholds (SMDs). Decreasing the threshold of Guio (2009) from 4+ to 2+ reduces the error by 2.5 million people, down to 0.3% of the adult population and 13.6% of the people in UHCNIR. This finding shows that (1) the validity of MDIs is sensitive to changes in thresholds and (2) validity is partially related to the level at which the thresholds are set. However, lowering the threshold might create other problems, possibly Type I errors, making this, at best, an *ad hoc* without much certainty about its effect on the overall error.

The contribution of this paper is threefold. The first relates to the methodology of validity evaluation. An appropriate selection and use of a partial criterion variable can help to identify the misclassification of certain groups and hence allows us to provide evidence on the empirical validity of poverty measures. Secondly, using this method, the paper provides empirical evidence on Type II errors in relation to a specific aspect of poverty—access to health care due to affordability problems. Thirdly, the paper explores the sources of error, which are embedded in the discussion on “conversion factors” and multidimensionality of poverty measurement.

Despite pointing to significant validity problems in the measurement of poverty arising from unmeasured health-related costs, the analysis does not provide a complete evaluation of empirical validity. There are two limitations of the proposed method based on a partial criterion variable. Firstly, such an analysis perforce focuses on a specific aspect of poverty. Hence, to gain a more comprehensive understanding of empirical validity, further research employing other partial criterion variables for different aspects of poverty is necessary. Secondly, and relatedly, only Type II errors are evaluated. As a result, the analysis provides only partial evidence on under or over-estimation of poverty numbers. In theory, the same method can be adapted to estimate the extent of Type I errors. These two issues, however, can best be the matters for future research.

 An important implication for poverty measurement arising from the analysis concerns the design of measures. Need differentials are not limited to health care. People also differ in their needs for education, transportation, child care or various other public or private goods and services that alter living conditions. Then, to account for the different experiences of poverty, MDIs as single scales constructed based on *some key* indicators might be insufficient. Employing more comprehensive equivalence scales or using measures including indicators from *each relevant dimension* (evaluated separately with a dimension-specific threshold before identifying poverty with an overall poverty threshold) might be the only way to adjust for varying need patterns. Yet, this option requires explicitly identifying the dimensions of poverty prior to their measurement. Only then, can aspects where adjustments for need differentials are necessary be comprehensively identified and relevant indicators for each dimension be devised. In other words, an elaborate theoretical definition that specifies the dimensional structure of poverty is a necessary condition for reaching empirically valid measures. In the interim, data limitations, however, are still the main concern.

## Figures and Tables

**Table 12:**
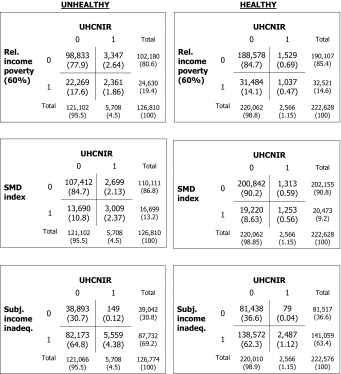
Cross tabulation of three poverty measures across health groups

## Appendix

See Tables 10, 11, 12.

**Table 10:** Risk Profiles for UHCNIR and poverty

	Total population	UHCNIR	EU2020
Gender			
Female	52.32	61.08	55.7
Age group			
15–24	12.75	7.07	15.49
25–44	34.36	29.28	31.42
45–64	31.98	39.36	32.84
65+	20.91	24.29	20.25
HH type			
Single person	15.4	22.08	22.22
2+ adults 0 child	43.4	38.54	35.58
Single parent	2.93	4.42	5.88
2 adults 1+ child	26.84	18.71	23.37
Extended family	11.2	14.79	12.63
Other	0.25	0.46	0.32
Marital status			
Never married	29.58	22.16	34
Married	54.56	49.43	44.42
Divorced/separated	15.87	28.41	21.58
Degree of urbanization			
Densely	49.38	44.18	45.4
Intermediate	26.24	21.63	22.64
Thinly	24.38	34.19	31.97
Full-time	42.66	24.77	22.5
Part-time	7.93	7.04	6.8
Economic status			
Unemployed	6.09	14.45	14
Student/trainee	7.38	2.56	9.65
Retired	23.59	28.4	23.55
Disabled	2.8	7.09	6.94
Domestic unpaid worker	7.24	10.83	12.15
Other inactive	2.31	4.87	4.4
Occupation			
Managers/senior officials	6.76	2.74	4.28
Professionals	12.2	4.16	4.8
Technicians/ass. professionals	15.59	8.35	9.17
Clerks	11.68	7.91	8.6
Service workers	12.92	14.44	14.8
Skilled agr. and fishery	5.39	12.92	10.95
Craft trade workers	14.19	17.93	17.14
Plant/machine operators	8.48	9.37	9.42
Elementary occupations	12.23	21.91	20.56
Army	0.57	0.28	0.29
Subj. health			
Bad	9.8	34.68	16.51
Chronic health problem			
Yes	30.93	56.11	36.61
Disability			
Yes	25.28	56.73	33.53

**Table 11:** Is UHCNIR a significant predictor of poverty?

	Poverty (eu2020) or/se
UHCNIR	3.055*** (0.09)
Log equiv. HH income	0.222*** (0.00)
Female	1.155*** (0.01)
Age	
15–24	0.842*** (0.01)
25–44	1.000 (.)
45–64	1.218*** (0.02)
65–80	0.813*** (0.01)
80+	0.893*** (0.02)
Marital status	
Never married	1.000 (.)
Married	0.495*** (0.01)
Separated	1.350*** (0.05)
Widowed	0.611*** (0.01)
Divorced	0.944** (0.02)
Subjective health	1.194*** (0.02)
Disability	1.460*** (0.02)
Chronic Health Problem	1.141*** (0.02)
N	349,438

Exponentiated coefficients

* *p* < 0.05; ** *p* < 0.01; *** *p* < 0.001

## Footnotes

1 Empirical validity is different from theoretical validity. *Theoretical validity* assesses whether the “reality” of poverty is accurately translated into a specified concept, therefore involves a conceptual debate regarding the ontological and epistemological positions on what poverty actually means rather than how the concept is operationalized. See Bollen (1989); Sireci (1998); Adcock and Collier (2001); Newton (2012); Newton and Shaw (2014) for discussions on the definition and types of validity.

2 Type I errors occur when a poverty measure identifies some people who are not actually in poverty. Type II errors arise when a poverty measure do not identify some people who are actually in poverty. See Calvo and Fernandez (2012) for a similar approach.

3 The analysis here focuses on the partial criterion variables that can assess only Type II errors. So, the partial criterion variable identifies the poor individuals, and its mismatch with a poverty measure shows the people who are missed by the measure despite being poor. Some other partial criterion variables can also be used to assess Type I errors. Such a criterion variable needs to be a yardstick for the non-poverty experience, and its mismatch with a poverty measure would then show the people who are wrongly identified as in poverty by the poverty measure.

4 This assumption is conceptually and empirically examined in the following sections.

5 For the absolute income measures, the calculation of thresholds does not include consumption of health care. Indeed, the error in the formal US poverty measure related to health differences are reported long ago by the classic paper of Mayer and Jencks (1989). For the relative income poverty measures, there is no adjustment for health care differentials, and theoretical ground for the threshold levels is generally contentious (e.g. Besharov and Couch 2012).

6 Given the information is collected only for adults, individuals aged below 16 are excluded.

7 The results for the overall EU might seem to be unaffected by this exclusion, given the similar results of the analysis with and without the HRPs’ of these countries. This is likely due to high sample size as well as the fact that the UHCNIR problem is generally really low in these countries.

8 There are yet further non-response problems (between 8 and 16%) for the health indicators in some countries such as Czech Republic, the UK, Poland, Estonia and Lithuania. Given the focus is on the EU, these missings are excluded.

9 This is equivalent to running a logistic regression where UHCNIR is regressed on the poverty measures.

10 UHCNIR is a binary variable so a logit transformation of UHCNIR is modeled. The coefficients are exponentiated so used in odds ratio forms. Interaction terms in odds term show multiplicative effects rather than marginal effects. Multiplicative interaction term controls for the baseline odds of healthy versus unhealthy on UHCNIR (see Buis 2010).

11
http://www.poverty.ac.uk/system/files/poverty/eurobarometer_heatmap.html.

12 The Health at Glance series of OECD periodically report the level of UHCNIR based on the data from EU-SILC. As shown by their latest study, the EU 27 average level of UHCNIR has been in decline between 2005 and 2009, from around 4% to around 2%, and then it has been stable in the period of 2009–2012 (OECD 2014). However, this rate varies between low and high income groups. For the low income groups, the rate has been around 8% in 2005, decreased to 4% in 2010 and slightly increased to 4.5% in 2012, while the rate for the high income groups has been around 1% for the whole period between 2005 and 2012 (OECD 2014). The UHCNIR rates also considerably differ among EU (21) countries. The highest prevalence is around 7.5%, for example, in Romania, Latvia and Bulgaria. Yet in countries such as the UK, Czech Republic and Spain, the problem is less than 0.3%.

13 Source: Eurostat http://appsso.eurostat.ec.europa.eu/nui/show.do.

14
http://ec.europa.eu/eurostat/tgm/table.do?tab=table&init=1&language=en&pcode=tps00001&plugin=1.

15 The relative validity of the MDIs can also be examined from this table. *Firstly*, the new versions of MDIs do not seem to improve the measurement performance compared to the old versions. This is likely because neither indices include an item related to access to health care. *Secondly*, indices with the lower thresholds perform better (MDs vs. SMDs). This result is also expected because higher thresholds constraint the number of people who falls below the cut-off. The higher the level of deprivation thresholds the lower the chance of identifying people with an UHCNIR problem. *Thirdly*, given the focus of consistent poverty measures on the intersection and more intense poverty experiences, the consistent poverty measures perform worse compared to the consensual measures.

16 The items are (1) serious difficulty meeting ends meet (form I), (2) feeling heavy burden of housing cost, (3) feeling heavy burden of debt, (4) having arrears for rent, mortgage or utility bills, (5) not being able to face unexpected expenses. The Cronbach alpha for the scale is 0.69. The index score is sum of deprived items (all binary).

17 The items are ability to afford to (1) spare money for oneself, (2) leisure activities, (3) drink/meal out with friends monthly, (4) replace worn-out furniture, (5) yearly go on a holiday. The Cronbach alpha for the scale is 0.77. The index score is sum of deprived items (all binary).

18 For the analyses in this section, only the SMD index is used but the results are similar for other MDIs.

## References

[CR1] Adcock R, Collier D (2001). Measurement validity: A shared standard for qualitative and quantitative research. American Political Science Review.

[CR2] Alkire S, Santos ME (2013). A multidimensional approach: poverty measurement beyond. Social indicators research.

[CR3] Alkire S, Foster J, Seth S, Santos ME, Roche JM, Ballon P (2015). Multidimensional poverty measurement and analysis.

[CR4] Allin S, Grignon M, Le Grand J (2010). Subjective unmet need and utilization of health care services in Canada: What are the equity implications?. Social Science and Medicine.

[CR5] Allin S, Masseria C (2009). Unmet need as an indicator of health care access. Eurohealth.

[CR6] Allin, S., & Masseria, C. (2012). Measuring access to health care in Europe. In A. McGuire and J. Costa-Font (Eds.), *The LSE Companion to Health Policy*. Cheltenham: Edward Elgar Publishing.

[CR7] Berthoud R, Bryan M (2011). Income, deprivation and poverty: A longitudinal analysis. Journal of Social Policy.

[CR8] Besharov DJ, Couch KA (2012). Counting the poor: New thinking about European poverty measures and lessons for the United States.

[CR9] Betti, G., & Lemmi, A. (2013). *Poverty and social exclusion: New methods of analysis.* New York: Routledge.

[CR10] Bollen KA (1989). Structural equations with latent variables.

[CR11] Bremer, P. (2014). Forgone care and financial burden due to out-of-pocket payments within the German health care system. *Health economics review*, *4*(1). 10.1186/s13561-014-0036-0 PMC4502068 26208936

[CR12] Buis ML (2010). Stata tip 87: Interpretation of interactions in non-linear models. The Stata Journal.

[CR13] Callan T, Nolan B, Whelan CT (1993). Resources, deprivation and the measurement of poverty. Journal of Social Policy.

[CR14] Calvo, C., & Fernandez, F. (2012). *Measurement errors and multidimensional poverty*. Retrieved from http://www3.qeh.ox.ac.uk/pdf/ophiwp/OPHIWP050.pdf.

[CR15] Chiappero-Martinetti E, Von Jacobi N (2011). Light and shade of multidimensional indexes: How methodological choices impact on empirical results. Quality of life in Italy: Research and Reflections. Social Indicators Research Series.

[CR16] Devaux, M., & De Looper, M. (2012). Income-related inequalities in health service utilisation in 19 OECD countries, 2008–2009. In *OECD health working paper series*, No. 58, OECD publishing.

[CR17] Dickes P, Fusco A, Marlier E (2010). Structure of national perceptions of social needs across EU countries. Social Indicators Research.

[CR18] Doorslaer EV, Koolman X, Jones AM (2004). Explaining income-related inequalities in doctor utilisation in Europe. Health Economics.

[CR19] Edin, K., & Lein, L. (1997). *Making ends meet: How single mothers survive welfare and low*-*wage work.* New York: Russell Sage Foundation.

[CR20] Gordon D (2006). The concept and measurement of poverty. Poverty and social exclusion in Britain. The Millennium Survey.

[CR21] Guio, A.-C. (2009). What can be learned from deprivation indicators in Europe. *Indicator subgroup of the Social Protection Committee*, *10*.

[CR22] Guio A-C, Gordon D, Marlier E (2012). Measuring material deprivation in the EU: Indicators for the whole population and child-specific indicators.

[CR23] Guio, A.-C., & Marlier, E. (2013). Alternative vs. current measures of material deprivation at EU level: What differences does it make? In *Luxembourg Institute of Socio*-*Economic Research (LISER) working paper series* 29.

[CR24] Halleröd B, Larsson D (2008). Poverty, welfare problems and social exclusion. International Journal of Social Welfare.

[CR25] Hick R (2014). On ‘consistent’ poverty. Social Indicators Research.

[CR26] Hick R (2016). Material poverty and multiple deprivation in Britain: the distinctiveness of multidimensional assessment. Journal of Public Policy.

[CR27] Huber, M., Stanciole, A., Wahlbeck, K., Tamsma, N., Torres, F., Jelfs, E., & Bremner, J. (2008). Quality in and equality of access to healthcare services: European Commission Directorate-General for Employment, Social Affairs and Equal Opportunities.

[CR28] Kakwani N, Silber J (2008). Quantitative approaches to multidimensional poverty measurement.

[CR29] Koolman, X. (2007). Unmet need for health care in Europe. In: *Comparative EU statistics on income and living conditions: Issues and challenges.* Proceedings of the EU-SILC conference, Eurostat, Helsinki.

[CR30] Layte R, Whelan CT, Maître B, Nolan B (2001). Explaining levels of deprivation in the European Union. Acta Sociologica.

[CR31] Litwin H, Sapir EV (2009). Forgone health care due to cost among older adults in European countries and in Israel. European Journal of Ageing.

[CR32] Lostao L, Regidor E, Geyer S, Aïach P (2007). Patient cost sharing and social inequalities in access to health care in three western European countries. Social Science and Medicine.

[CR33] Mack J, Lansley S (1985). Poor Britain.

[CR34] Maître, B., Nolan, B., & Whelan, C. T. (2012). Reassessing the EU 2020 poverty target an analysis of EU-SILC 2009. In *UCD Geary Institute Discussion Paper Series.*

[CR35] Mayer SE, Jencks C (1989). Poverty and the distribution of material hardship. Journal of Human Resources.

[CR36] Morciano M, Hancock R, Pudney S (2015). Disability costs and equivalence scales in the older population in Great Britain. Review of Income and Wealth.

[CR37] Newton PE (2012). Clarifying the consensus definition of validity. Measurement: Interdisciplinary Research & Perspective.

[CR38] Newton, P., & Shaw, S. (2014). *Validity in educational and psychological assessment.* London: Sage.

[CR39] Nolan, B., & Whelan, C. T. (1996). Resources, deprivation, and poverty. *OUP Catalogue*. Oxford

[CR40] Nolan, B., & Whelan, C. T. (2007). On the multidimensionality of poverty and social exclusion. In *Inequality and poverty re*-*examined*, (Part II), pp. 146–165.

[CR41] Nolan, B., & Whelan, C. T. (2011). Poverty and deprivation in Europe. *OUP Catalogue*. Oxford

[CR42] OECD. (2014). *Health at a Glance: Europe 2014.* OECD Publishing. doi:10.1787/health_glance_eur-2014-en

[CR43] Or, Z., Jusot, F., & Yilmaz, E. (2008). *Impact of health care system on socioeconomic inequalities in doctor use.* IRDES.

[CR44] Ravallion M (2011). Mashup indices of development. The World Bank Research Observer.

[CR45] Ringen S (1985). Toward a third stage in the measurement of poverty. Acta Sociologica.

[CR46] Ringen S (1988). Direct and indirect measures of poverty. Journal of Social Policy.

[CR47] Sen A (1992). Inequality reexamined.

[CR48] Sireci SG (1998). The construct of content validity. Social Indicators Research.

[CR49] Townsend P (1979). Poverty in the United Kingdom: A survey of household resources and standards of living.

[CR50] Whelan CT (2007). Understanding the implications of choice of deprivation index for measuring consistent poverty in Ireland. Economic and Social Review.

[CR51] Whelan CT, Layte R, Maître B, Nolan B (2001). Income, deprivation, and economic strain. An analysis of the European community household panel. European Sociological Review.

[CR52] Whelan CT, Maître B (2007). Income, deprivation and economic stress in the enlarged European Union. Social Indicators Research.

[CR53] Whelan CT, Maître B (2012). Understanding material deprivation: A comparative European analysis. Research in Social Stratification and Mobility.

[CR54] Whelan CT, Maître B (2013). Material deprivation, economic stress, and reference groups in Europe: An analysis of EU-SILC 2009. European Sociological Review.

[CR55] Whelan CT, Nolan B, Maître B (2014). Multidimensional poverty measurement in Europe: An application of the adjusted headcount approach. Journal of European Social Policy.

[CR56] Zaidi A, Burchardt T (2005). Comparing incomes when needs differ: Equivalization for the extra costs of disability in the UK. Review of Income and Wealth.

